# A computational framework for gene regulatory network inference that combines multiple methods and datasets

**DOI:** 10.1186/1752-0509-5-52

**Published:** 2011-04-13

**Authors:** Rita Gupta, Anna Stincone, Philipp Antczak, Sarah Durant, Roy Bicknell, Andreas Bikfalvi, Francesco Falciani

**Affiliations:** 1 School of Biosciences, University of Birmingham, Edgbaston, Birmingham B15 2TT, UK; 2 Institute of Biomedical Research, Medical School, University of Birmingham, Edgbaston, Birmingham B15 2TT, UK; 3 INSERM E 0113, Molecular Angiogenesis Laboratory, Université de Bordeaux 1, 33405 Talence, France

## Abstract

**Background:**

Reverse engineering in systems biology entails inference of gene regulatory networks from observational data. This data typically include gene expression measurements of wild type and mutant cells in response to a given stimulus. It has been shown that when more than one type of experiment is used in the network inference process the accuracy is higher. Therefore the development of generally applicable and effective methodologies that embed multiple sources of information in a single computational framework is a worthwhile objective.

**Results:**

This paper presents a new method for network inference, which uses multi-objective optimisation (MOO) to integrate multiple inference methods and experiments. We illustrate the potential of the methodology by combining ODE and correlation-based network inference procedures as well as time course and gene inactivation experiments. Here we show that our methodology is effective for a wide spectrum of data sets and method integration strategies.

**Conclusions:**

The approach we present in this paper is flexible and can be used in any scenario that benefits from integration of multiple sources of information and modelling procedures in the inference process. Moreover, the application of this method to two case studies representative of bacteria and vertebrate systems has shown potential in identifying key regulators of important biological processes.

## Background

In the last ten years the development of functional genomics technologies has provided us with the ability to generate quantitative data representing the molecular state of cells and tissues at a genome level [1,2]. These datasets can be in the form of a time series representing the dynamics of gene expression profiles (e.g. mRNA, proteins and metabolites) in response to a given stimulus, such as an environmental perturbation, the effect of a growth factor or an experimentally induced gene deletion. Despite the relatively large amount of information, predicting underlying regulatory networks from observational data is still not trivial and is a matter of intense research [3].

A number of reverse-engineering approaches have been proposed. Some of these are designed to infer networks from a compendium of perturbation experiments while others are able to use time course data to develop dynamical models of gene interaction. Bayesian networks have been among the first to be applied to biological problems [4]. They work by inferring probabilistic relationships between variables, can use either time course or steady state data and allow integration of prior knowledge in the model. Correlation-based methods [5,6] compute correlation coefficients between variables to infer the underlying network topology. State-space models (SSMs) [7,8], and ODE-based methods [9,10], on the other hand use time-course data to develop dynamic models of gene regulatory networks (GRN). For an extensive overview of these methodologies see: [11,12].

The general validity of the principal of integrating multiple data sources in the reverse-engineering process is exemplified by the observation that the best performing methods utilize some degree of integration between different experiments [13]. For example, the top performing method in the third edition of the "Dialogue for Reverse Engineering Assessments and Methods" (DREAM), developed by Yip et al. [14], was based on a combination of a statistical error-model and ODE modeling to integrate gene knock-out (KO) and time-course experiments. Interestingly, Yip et al. [14] also noted that a relatively simple differential gene-expression analysis, comparing wild-type and mutant strains, was in itself a very good representation of the underlying gene regulatory network. However, not all KO experiments are likely to be equally informative and identifying a priori the most relevant genes is not a trivial task. Moreover, large-scale gene-inactivation experiments are not a viable option for many non-model species.

Therefore, there is the need to expand the repertoire of available network inference tools by developing more methods that allow integration of multiple data sources and have the flexibility to use a wide range of datasets and information. In order to achieve this objective, we set out to develop a computational framework that has the potential to combine different inference methodologies, multiple datasets, as well as any pre-existing biological knowledge. We based this approach on an ODE framework combined to a multi-objective optimization (MOO) procedure for parameter estimation. We named this method "*Network-Inference with Multi Objective Optimization*" (NIMOO).

## Methods

### The basic network inference framework: Model Equations and parameter estimation of a single objective optimization procedure

Gene interactions in a regulatory network can be modelled using a set of ordinary differential equations [9,10]. In this implementation we have used a linear ODE model where the interaction between genes is additive. In this context, changes in the expression of a given gene depend on a weighted linear sum of the expression of its regulators:(1)

where, x_i _represents expression level for gene *i*, *b_i _*represents the effect of the external perturbation on gene i, and, N is the number of genes in the dataset. The parameter matrix **w **is obtained by minimizing the Squared Error (*E^SQE^*)(2)

The gene regulatory network (GRN) is then inferred from the optimized parameter matrix **w**. The matrix element |w_ij_| indicates the strength of the interaction between genes *i *and *j *(with gene *j *regulating gene *i*), and, sign (w_ij_) indicates whether the effect is excitatory (w_ij _> 0) or inhibitory (w_ij _< 0)

In our implementation of single objective optimisation (SOO), minimisation of *E^SQE ^*was achieved using the trust-region method based on the interior-reflective Newton method [15,16]. In this method the minimisation process involves defining a trust region where the objective function *SQE *can be approximated with a simpler function *q*. For successive iterations, function *q*, in conjunction with the Preconditioned Conjugate Gradient Method [16], is used to find a new trust region where the function SQE is lower. The process is terminated when the change in function value is less than a pre-determined tolerance (10^-6^).

### Parameter estimation using a multi-objective optimization procedure

Multi-objective optimisation (MOO) is based on minimisation of *E^SQE ^*in conjunction to additional objective functions, *E^object^*, which are built as Euclidean distance between the parameter matrix **w **and objectives **O **constructed from additional data and/or existing knowledge:(3)

To implement multi-objective optimization we have used the goal attainment method [17,18]. In this method the problem of simultaneously optimizing multiple functions is reduced to the task of standard minimization. A set of *goal*s *[J_1_, J_2_, ...,J_m_] *and weights [*θ_1_, θ_2_, ..., θ_m_] *are assigned to the objective functions *F = [F_1_, F_2_, ..., F_m_*], where, *F_1 _= E^SQE^, F_2 _= E^Object ^*etc. Also, a scalar dummy variable γ is introduced so that the aim is to minimize for γ such that(4)

The term *θ_k_γ *introduces flexibility in the degree of goals attained. Also, the weight factor θ_k _can be used to assign relative importance to the objectives: Thus, from Equation 4, θ*_k _= 0 *implies hard goal for the corresponding objective function *F_k_*. For all results presented in this paper, unless mentioned otherwise, the goal and weight corresponding to the objective SQE were set to 0.

### Overall strategy for the development of a MOO-based inference method

**Figure 1:**
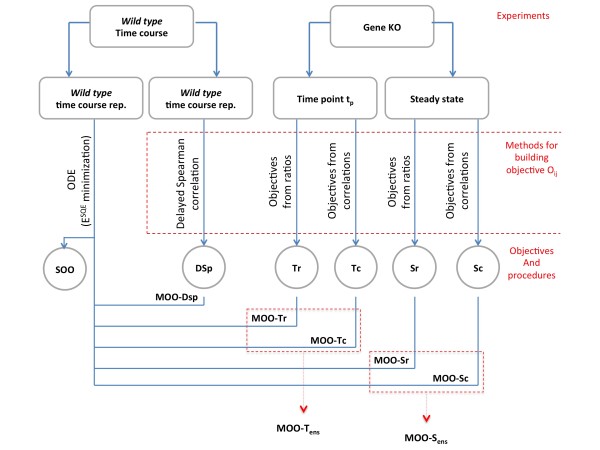
**Overview of the NIMOO methodology**. The figure shows in a schematic format the relationships between type of experiment, methods used to build the O_ij _objectives and the MOO procedures. Details are given in the Methods section.

Figure 1 shows in a schematic format the different procedures that are part of the NIMOO framework and their relationships with the experimental datasets.

The principle behind NIMOO, as detailed in the above sections, is to infer the gene regulatory matrix (GRM) **w **by minimizing the *E^SQE ^*for the ODE system in conjunction with additional objective functions, *E^object^*, which represent the distance between the parameter matrix w_ij _and objectives O_ij _constructed from additional information.

In principal, objectives O_ij _can be constructed from any data available on the underlying regulatory network. In this paper, we focused on two possible scenarios.

In the first case, we considered the possibility that MOO might be used to integrate two different network-inference procedures, for example applied to independent replicates of a time-course experiment. In this implementation we used time-delayed Spearman rank-correlation [6] to develop a matrix O_ij _(**Equation 3**) representing the degree of statistical correlation from any pair of genes within a set time delay interval (Figure 1, objective DSp).

Alternatively, *O_ij _*can be built from the results of gene inactivation experiments. We reasoned that these experiments might fall in at least two categories. In the first case the gene is deleted at some stage of the lifecycle of the organism so that gene expression measurements can only be acquired after the new steady state has been reached. This can be easily achieved, by a plethora of gene knockouts (KOs) methodologies in model systems ranging from *E. coli *to mice. Alternatively, gene inactivation could be achieved by using biochemical inhibitors or RNA interference. In this scenario mRNA expression can be monitored at different time points following intervention. In the context of MOO both scenarios lead to a gene-expression matrices where rows are represented by genes and columns by gene KO experiments. From each of these matrices an objective O_ij _can be computed to represent the relationship between every gene pair (Figure 1, objectives Tc, Tr are derived from expression data at a given time-point shortly after gene inactivation whereas objectives Sc and Sr derive from expression data at a single time point at steady state; for further details on how to compute O_ij _see sections below).

Different procedures may be used in combination using an ensemble approach; in this paper we describe the results of combining MOO-Tr with MOO-Tc (Figure 1, MOO-T_ens_) and MOO-Sr with MOO-Sc (Figure 1, MOO-S_ens_).

All MOO procedures developed within NIMOO have been compared with the accuracy of an ODE model developed by minimizing E^SQE^, a procedure that we called single-objective optimization (Figure 1, SOO).

The paragraphs below describe in detail how the different objectives were computed.

### Construction of a time-delay correlation matrix (objective DSp)

To test the potential of MOO to combine different network inference approaches we choose to build an objective based on time-delayed Spearman Rank-correlation [6] (Figure 1, objective DSp). DSp was computed as follows: For each gene pair (*i,j*), the expression profile of gene *i *is shifted along the time axis with respect to that of gene *j*. The Spearman Rank-correlation coefficient is calculated for varying time delays and the largest coefficient from this list forms the (*i,j*)^th ^element of the delayed Spearman Rank-correlation matrix *d-SRC*. We also construct a time delay matrix dt from the corresponding values. The objective DSp is then obtained from *d-SRC *by equating all *d-SRC(i,j) = 0 *for which *dt(i,j) < t_o_*, so that only gene pairs with delay of t_o _or more are considered.

### Construction of a gene KO matrix: a ratios-based procedure

The objectives Tr and Sr (Figure 1) were constructed by computing the ratios between the expressions of each gene *i *in the mutant *j *and the expression of gene *i *in the wild type. The expression of gene *i *is taken either at a given time point t_p _after inactivation (Tr) or at the steady state (Sr). We selected t_p _as the time point where the largest numbers of genes have the highest derivative in absolute value. We found that this heuristic rule allowed us to identify a value of t_p_, which often (8 out of 9 networks tested) corresponded to the highest AUC values within a tolerance of 5% (Figure S1).

### Construction of a gene KO matrix: a correlation-based procedure

The objectives Tc and Sc (Figure 1) were computed by calculating the correlation between the expressions of every pair of genes (gene *i*, gene *j*) across all KO samples. Similarly to the ratio procedure, the Tc matrix was built using the measure of gene ***i ***expression at time **t_p_**, where **t_p _**was chosen as detailed above.

### Combining MOO procedures using an ensemble approach

The ratio and correlation methods were integrated to produce a single model by using an ensemble approach. Within this procedure, a GRM **w_a _**was constructed so that |w_a_(i,j)| = |w_r _(i,j)| and sign(w_a_(i,j)) = sign(w_c _(i,j); Where **w_r _**and **w_c _**represent two GRMs obtained from the ratio and correlation procedures, respectively. As exemplified in Figure 1, MOO-S_ens _represent the result of combining the MOO-Sr and MOO-Sc procedures whereas MOO-T_ens_, is the result of combining the MOO-Tr and MOO-Tc procedures.

### Simulated data

The validation study has been performed using the java application GeneNetWeaver (GNW) http://gnw.sourceforge.net[19]. This network generator has been used as part of the DREAM Initiative [20]. It builds synthetic networks by specifying a biologically relevant topology and implementing an ODE model to generate synthetic data. GNW grows the initial topology from a seed node (selected randomly) in a *Source Gene Network *(*E. Coli *in this application) by progressively adding a randomly selected neighbouring node till the desired size is reached. Each model can be used to generate simulated time course gene expression data either with the intact network or following deletion of one of the nodes.

We tested the performance of MOO in conjunction with the objectives D-Sp (MOO-Sp), Tc (MOO-Tc), Tr (MOO-Tr), Sc (MOO-Sc) and Sr (MOO-Sr). Each of these procedures was applied to ten independent networks of size 20, 35 and 50 genes. The gene KOs dataset associated with every network was build by generating synthetic data after the stepwise deletion of each gene in the network.

All GNW-generated network-models were used to simulate time-series datasets (26 time points, t_max = 200) as well as steady-state data for all KOs.

### Data processing and optimisation procedure

Noise was added to the simulated data (5% of the signal) to mimic variability in experimental replication. Polynomial fitting was used for interpolation of the time-series data [21] after averaging three experimental replicates. 200 interpolated, equally spaced time-points were then computed and used as input of the MOO procedures. Optimisation of the matrix **w **was initiated from a randomly generated matrix. In order to test the reproducibility of the optimization methods, fifty independent runs of optimization from each MOO procedure were carried out for a subset of the GNW networks. We found that the AUCs values were always within 0.2%.

### Network inference accuracy

In order to evaluate various MOO methods we compared the inferred gene-regulatory matrix **w **with the true network topologies. The accuracy of the inference process for undirected networks was quantified by using the area under curve (AUC) of a ROC plot (False Positive Rate (FPR) versus True Positive Rate (TPA)). For direct-signed networks the AUC was computed by plotting TNR (True Negative Rate) versus TPR as described in [10]. The distribution of AUC values for boxplots and these represented each batch of networks were compared when appropriate using a Wilcoxon's non-parametric rank sum test [22].

### Modelling *in vivo *tumour development

In order to assess the potential of NIMOO to model true biological systems we have used two microarray datasets generated in our laboratory.

We first used an in vivo model of glioblastoma development to test the MOO-Sp procedure. In this experimental model [23] U87 human glioma cells (ATCC, USA) were maintained in DMEM with 10% FBS, antibiotics, and l-glutamine. Fertilized chicken eggs (*Gallus gallus*; E.A.R.L. Morizeau, Dangers, France) were incubated at 37°C and 80% humidified atmosphere. On day 4 of development, a window was made in the eggshell after punctuating the air chamber and sealed with Durapore tape. On embryonic day 10, a plastic ring was placed on the embryo chorioallantoic membrane (CAM), and 3 million to 5 million U87 cells in 20 μl of medium were deposited after gentle laceration of surface. Implantation experiments were performed in triplicate, and, from day 11 to day 15, mRNA from the growing tumour was extracted every 12 hours using the standard protocol provided in the Qiagen RNeasy kit. Labelling was performed using protocol V5.7 provided in Agilent's Quick Amp One-Colour labelling kit and hybridized onto human Agilent microarrays (AMADID:014850). Data were normalized using quantile normalization and genes differentially expressed over time were identified using the statistical methodology SAM [24]. 58 genes were selected among the most differentially expressed across the time course (Table S3) and used as input of the modelling procedure.

### Modelling *E. coli *acid stress

In order to fully test the potential of MOO methodology we have applied the MOO-S_ens _procedure to model the *E. coli *response to mild acid conditions, a stress signal relevant to pathogenesis in diarrheagenic *E. coli *strains [25]. The procedure was used to integrate two microarray datasets representing the dynamic response of the *E. coli *MG1655 strain to acid exposure (pH = 5.5) and a gene KO experiment performed in the related strain *E. coli *BW 25113, representing the transcriptional state of strains mutated in the 26 most differentially expressed genes. In this analysis we aim to reverse engineer the interactions between these 26 genes. The time-course analysis of the response of the *E. coli *strain MG1655 to acid exposure was performed maintaining a constant cell number (OD_600 nm _= 2) using a media replenishment procedure. Samples were collected every 5 minutes for 1 hour in three replicated experiments. Mutant strains representing 26 of the most differentially regulated genes over time were selected from the BW25113 KEIO mutant collection [26] and analysed using microarrays as described below. Experiments were performed exactly in the same conditions as the MG1655 strain but only control and 15 minutes in acid were processed for microarray analysis.

Microarray analysis was performed as follows. 10 ml of cultures were samples at the different time points and stabilized by adding a solution of phenol-ethanol (final concentration of 19% phenol and 1% ethanol). Cell pellets were recovered by centrifugation and stored at -80°C. mRNA was extracted using the standard protocol provided in the Quiagen RNEasy kit (QUIAGEN, USA) and labelled with Cy5 labelled dCTP (Amersham Biosciences, USA) using the CyScribe Post-Labelling Kit (Amersham Biosciences, USA). Probes were then purified using CyScribe purification Kit (Amersham Biosciences, USA) according to the manufacturer's instructions. Labelled probes (80 pmol) were then hybridized on Operon *E. coli *Ultra GAPS microarray slides (Corning, USA). Slides were washed in AdvaWash automated washing station (Adavlytix, USA) and scanned with the ScanArray^® ^GX (PerkinElmer^®^, USA), using the ScanArray^® ^software. Data were normalized using quantile normalization and genes differentially expressed over time were identified using the statistical methodology SAM [25]. We modelled the *E. coli *datasets by using the ensemble approach integrating both correlation and ratio procedures as described above. In order to generate comparable sparse networks we thresholded the connectivity matrix **w **to obtain networks with same number of genes in the networks (25).

### Method implementation and datasets availability

NIMOO was implemented in MATLAB [27]. Code and datasets are available at this URL: http://biptemp.bham.ac.uk/NI_MOO/NI_MOO.zip.

## Results

### Combining different inference methodologies within the MOO framework improves the accuracy of network reconstruction

The first scenario we considered involved combining two network inference methods to model replicated time course experiments. To achieve this, we used delayed Spearman Rank-correlation [6] to build the objective O_ij _(**Equation 3**) for MOO.

**Figure 2:**
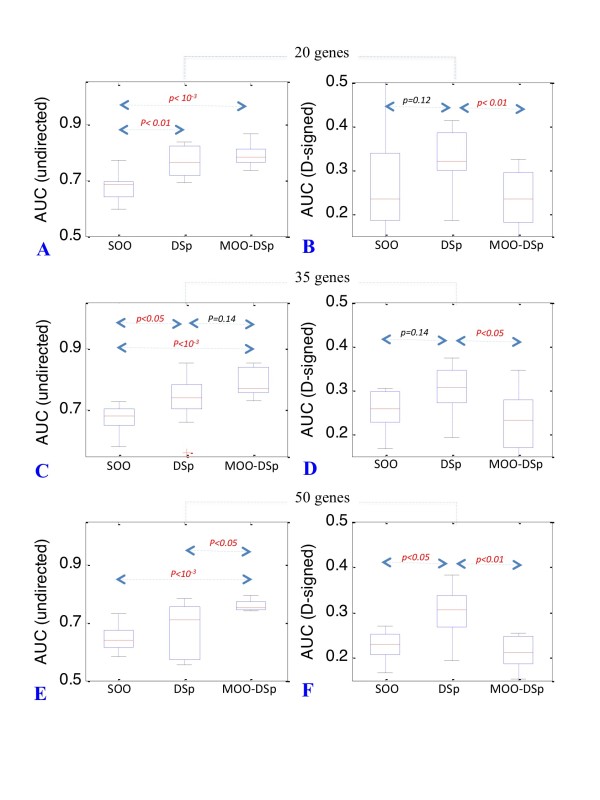
**Distribution of AUC values for the MOO-Sp procedure**. Boxplots representing the distribution of AUC values for 20, 35 and 50-gene networks. Accuracy of GRN reconstruction for both undirected (panels A, C and E) and directed-signed (panels B, D and F) networks is given for the SOO, D-Sp and MOO-dSp procedures. *p values *are indicated in red when significant (*α = 0.05*). Borderline *p values *and indicated in black (*α = 0.2*).

We discovered that the simple time-delayed correlation matrix DSp (Figure 1) was more effective than SOO to reverse engineer undirected networks of size 20 and 35 (up to 10% increase, *p < 0.05) *(Figure 2A, C and Table 1). The MOO-DSp procedure was always more effective than SOO (up to 11% increase, *p < 10^-3^*) (Figure 2A, C, D and Table 1) and gave higher AUC values than the simple DSp matrix for networks of size 50 (8% increase, *p < 0.05*) (Figure 2D and Table 1). With directed-signed networks the d-SP matrix was more effective than SOO although *p values *were borderline except for the larger 50 genes network size (7% increase, *p < 0.01*) (Figure 2B, D, F and Table 1).

**Table 1:** Accuracy of GRN inference with MOO-dSp

Type	Size	SOO	D Sp	MOO dSp
**Undirected**	20	0.68	0.77	0.79
**Undirected**	35	0.68	0.73	0.79
**Undirected**	50	0.65	0.68	0.76
**Directed-signed**	20	0.27	0.32	0.23
**Directed-signed**	35	0.27	0.30	0.22
**Directed-signed**	50	0.24	0.30	0.31

The table shows the average AUC values obtained for 20, 35 and 50-gene networks, for undirected and directed-signed networks, with the SOO, D-Sp and MOO-dSp procedures.

Overall, we can conclude that in the event that only replicated time-course experiments are available, a situation which is not uncommon, the integration between two methodologies can lead to a dynamical model with better accuracy than one solely based on a SOO procedure.

### Integrating time-course and gene inactivation experiments within the MOO framework: A ratio-based procedure

Having shown that MOO is an effective approach to combine different network-inference methodologies we set to test whether it may also provide a solution to integrate time-course and gene-inactivation experiments.

**Figure 3:**
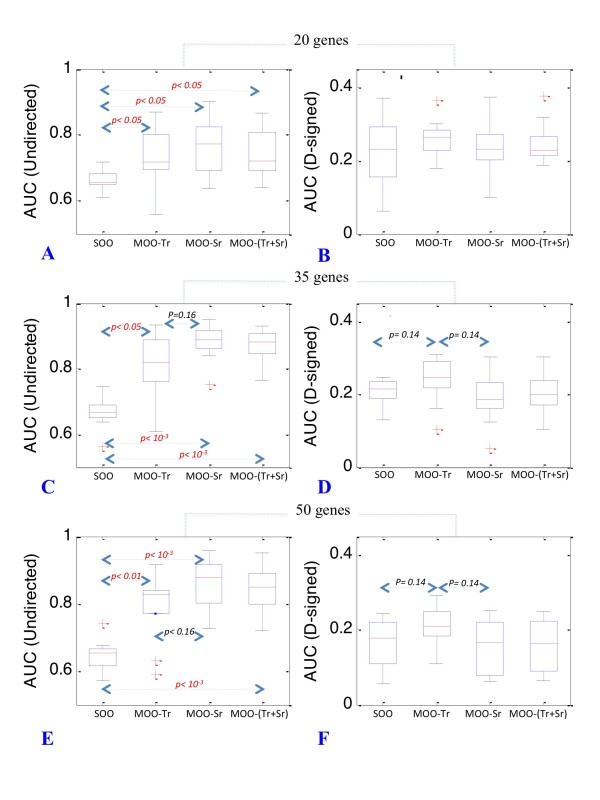
**Distribution of AUC values for ratio-based inference procedures**. Boxplots representing the distribution of AUC values for 20, 35 and 50-gene networks. Accuracy of GRN reconstruction for both undirected (panels A, C and E) and directed-signed (panels B, D and F) networks is given for the SOO, MOO-Tr, MOO-Sr, MOO-(Tr+Sr) procedures. *p values *are indicated in red when significant (*α = 0.05*). Borderline *p values *and indicated in black (*α = 0.2*).

We initially approached this challenge by applying the MOO-Sr and MOO-Tr procedures to simulated data, representing gene expression in KO experiments either at the steady state or at a single time point t_p _after inactivation. We discovered that AUC could vary considerably (up to 25%) depending on the value of t_p _(Figure S1 in Additional File 1), suggesting that the choice of the right time-point was an important factor. We also observed that the time point at which the largest number of gene expression profiles had the highest derivative often lead to higher AUC values within a tolerance of 5% (Figure S1 in Additional File 1). Although this has not to be considered a criterion to identify the optimal t_p _value we believe it represents a useful guideline. MOO substantially improved the prediction of undirected networks, with all network sizes tested. The largest gain we observed was a 20% increase respect to SOO with 35-gene networks, with the MOO-Sr procedure (*p < 10^-3^*) (Figures 3A, C, E, Table 1 and Table 2). Overall, the MOO-Sr procedure also gave consistently higher AUC values than MOO-Tr although *p values *were borderline significant (*p value *= 0.16). Combining ***T-r ***with ***S-r ***in the MOO procedure (MOO-(Tr+Sr)) did not further improve the accuracy of network inference (Figures 3A, C and 3E and Table 2). For direct-signed networks, only MOO-Tr gave consistent higher AUC values respect to SOO although *p values *were borderline significant (*p value *= 0.12) (Figures 3B, D, F and Table 2).

**Table 2:** Accuracy of GRN inference by integrating gene KO datasets in the MOO framework

Type	Size	Ratio methods MOO-Sr/MOO-Tr	Corr. methods MOO-Sc/MOO-Tc	Ensemble MOO S_ens_/MOO T_ens_
**Undirected**	20	**0.77/0.70**	0.70/0.65	0.77/0.70
**Undirected**	35	**0.88/0.79**	0.75/0.70	0.88/0.79
**Undirected**	50	**0.85/0.79**	0.75/0.69	0.85/0.79
**Directed-signed**	20	0.23/0.24	**0.42/0.36**	0.47/0.39
**Directed-signed**	35	0.18/0.21	**0.54/0.49**	0.69/0.57
**Directed-signed**	50	0.17/0.22	**0.54/0.45**	0.64/0.51

The table shows the AUC values obtained for 20, 35 and 50-gene networks, for undirected and direct-signed networks with MOO procedures integrating time course and gene KO datasets. Ratio, correlation and ensemble-based methods are shown in separate columns. AUC values for different procedures within each column are separated by a forward slash. Note that we marked the AUC values in bold to highlight the opposite trend in inference accuracy of the ratio and correlation procedures.

### Integrating time course and gene inactivation experiments within the MOO framework: A correlation-based procedure

In this section, we describe the results of the correlation-based procedure to construct MOO objectives from mutant gene expression data. As detailed in the methods section, this approach works by computing the correlation between the expression profiles of every pair of genes across the mutant samples.

**Figure 4:**
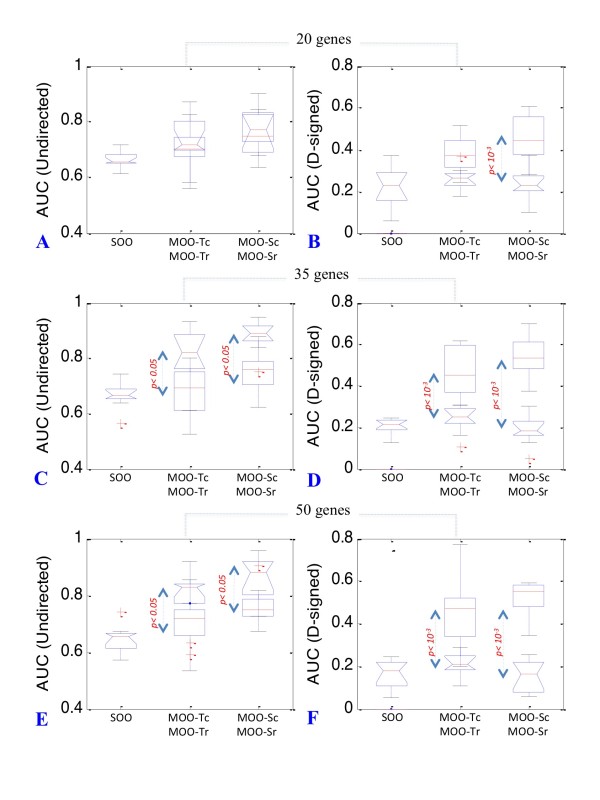
**Comparison of AUC values for ratio and correlation based inference procedures**. In this figure the distributions of AUC values for ratio and correlation-based methods are represented on the same plots for comparison. Accuracy of GRN reconstruction for both undirected (panels A, C and E) and directed-signed (panels B, D and F) networks is given for the SOO, MOO-Tc, MOO-Tr, MOO-Sc and MOO-Sr procedures. Distribution of AUC value for ratio-based procedures are represented by notched boxplots whereas these for correlations-based procedures are represented by rectangular boxplots. *p values *are indicated in red when significant (*α = 0.05*). Borderline *p values *and indicated in black (*α = 0.2*).

We discovered that inference accuracy of the ratio and correlation methods had opposite trends with respect to undirected and directed-signed networks. More precisely, the correlation-based objectives gave higher AUC values for direct-signed networks and lower AUC values than the ratio method for undirected networks. The differential in AUC values between the two methods was statistically significant for both undirected and directed-signed networks (up to 12% with undirected networks and up to 37% with directed-signed networks, *p < 0.05 *and *p < 10^-3 ^*respectively) (Figure 4 and Table 2) Interestingly, the largest differential corresponded to the directed-signed larger 50-gene networks (37%, *p < 10^-3^*).

**Figure 5:**
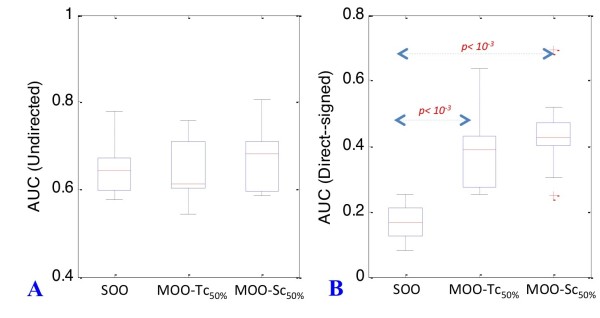
**MOO with incomplete gene KO datasets**. Boxplots representing the distribution of average AUC values for 50 gene undirected (panel A) and directed-signed (panel B) networks. Accuracy of GRN reconstruction is given for the SOO, MOO-Tc_50% _and MOO-Sc_50% _procedures. *p values *are indicated in red when significant (*α = 0.05*). Borderline *p values *and indicated in black (*α = 0.2*).

We discovered that the method of correlation is efficient even when a partial dataset is available. Figure 5 shows the results of the analysis for a 50-gene network when KO data is available for 50% of the genes. We did not observe any increase in inference accuracy for undirected networks with the MOO-Sc and MOO-Tc procedures (Figure 5A and Table 3). However, a considerable increase in accuracy was detected when inferring directed-signed networks (Figure 5A and Table 3, up to 27% improvement versus a SOO approach, *p < 10^-3^*).

**Table 3:** Accuracy of GRN inference with partial coverage gene KO datasets

Type	Size	SOO	MOO-Tc_50%_	MOO-Sc_50%_
**Undirected**	50	0.64	0.64	0.67
**Directed-signed**	50	0.17	0.38	0.44

The table shows the average AUC values obtained for 50-gene networks, for undirected and directed-signed networks, with the MOO-Tc_50% _and MOO-Sc_50% _procedures.

### Combining ratio and correlation-based procedures further improve inference acuracy

Since we have shown that correlation and ratio-based methods provide complementary information, we decided to test whether combining them using an ensemble approach could result in an even higher accuracy of the network inference process.

**Figure 6:**
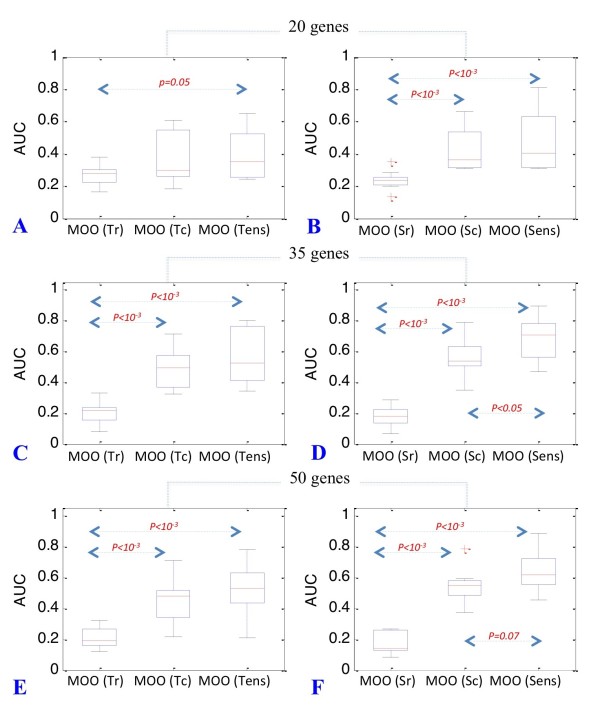
**Combining MOO procedures**. Boxplots representing the distribution of AUC values for 20, 35 and 50-gene networks obtained by the application of ensemble approach combining correlation and ratio-based MOO procedures. Accuracy of GRN reconstruction for directed-signed networks is given for the MOO-Tr, MOO-Tc, MOO-T_ens _procedures (panels A, C and E) and for the MOO-Sr, MOO-Sc, MOO-S_ens _procedures (panels B, D and F). *p values *are indicated in red when significant (*α = 0.05*). Borderline *p values *and indicated in black (*α = 0.2*). *p values *are indicated in red when significant (*α = 0.05*). Borderline *p values *and indicated in black (*α = 0.2*).

This approach was successful. AUC values for the ensemble models built from combining the MOO-Tr and MOO-Tc approaches (MOO-T_ens_) were comparable to the best performing MOOTc models (Figure 6A, C and 6E and Table 2) whereas models built from combining MOO-Sr and MOO-Sc (MOOS_ens_) yield even higher AUC values than MOO-Sc models for the larger 35 and 50-gene networks (15% and 10% increased AUC values, *p < 0.05*) (Figure 6D and 6F and Table 2).

Overall, MOO-S_ens _was the best performing procedure in inferring directed-signed networks. Therefore, we concluded that if both time-course and KO data are available for a sub-set of genes of interest, MOO-S_ens _may be the procedure of choice.

### Modelling biological systems with NIMOO

In order to test the validity of NIMOO to model real biological systems, we have analysed two datasets generated in our own laboratory. The first was a replicated gene-expression-profiling time-course experiment representing a model of *in vivo *glioblastoma development. A sub-set of these data were modelled with the MOO-Sp procedure. The second dataset included a time course representing the transcriptional response of *E. coli *during acid adaptation and the expression profiling of a compendium of 26 mutants exposed to acid. Because of the availability of both time-course and mutant steady-state data we applied the MOO-S_ens _procedure.

#### Modelling *in vivo *tumour development

**Figure 7:**
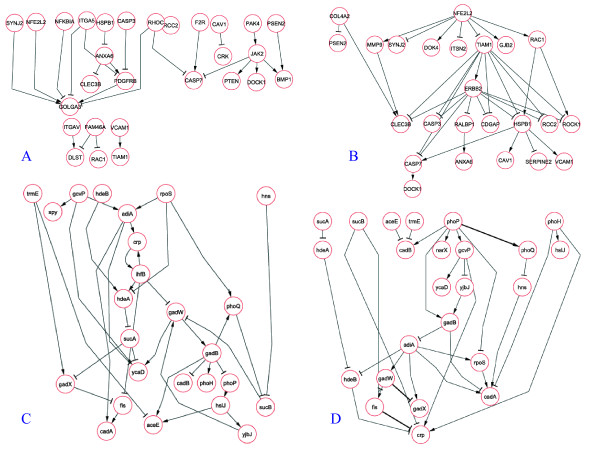
**Inference of of biologically relevant networks**. Gene regulatory networks obtained from the glioblastoma (panels A, B) and *E. coli *acid stress datasets (panels C and D). Networks obtained from SOO (panels A and C) and MOO (panels B and D) procedures are shown.

Our model identified a network organized around three main hubs (NFE2L2, ERBB2 and HSPB1) (Figure 7B). NFE2L2 (Nuclear factor E2 p45-related factor 2; commonly called Nrf2) is a transcription factor that binds to the cis-regulatory, antioxidant response element and transcriptionally up-regulate an environmental stress response program [28]. Nrf2 is critical for protection against a wide range of inflammatory conditions, hyperoxia, fibrosis, hepatotoxicity, carcinogenesis, neurodegeneration, cardiovascular disease and aging [29]. Inactivation of Nrf2 in some cancers, promote tumorigenicity and resistance to an array of chemotherapeutic compounds [30]. The biological role of Nrf2 as a master regulator of a crucial response is fully reflected in our model that identifies Nrf2 as the most upstream network node with the highest number of connections. Note that without the application of the MOO methodology this network feature was not inferred (Figure 7A).

The other network hubs are also known important signalling factors. ERBB2 is a gene that encode for a member of the epidermal growth factor (EGF) receptor family of receptor tyrosine kinases. This protein has no ligand-binding domain but it does bind tightly to other ligand-bound EGF receptor family members enhancing kinase-mediated activation of downstream signaling pathways. HSPB1has a cytoprotective function and support of cell survival under stress conditions. This gene is also involved in the apoptotic signalling pathway and interacts with actin and intermediate filaments to protect actin filaments from fragmentation. It also preserves the focal contacts fixed at the cell membrane. These observations support the hypothesis that Nrf2 sits high in the hierarchy of events leading to the development of a fully vascularized tumour.

#### Reverse engineering an *E coli *acid response network

Both single objective (SOO) and multiobjective (MOO) optimization methods were applied to investigate regulatory networks representative of *E. coli *acid adaptation. In order to test the full potential of the NIMOO methodology, we used both time-course and gene-inactivation experiments.

The networks identified using either the time course data (SOO procedure) or the combination of time course and gene KO profiles (MOO procedure) are represented in Figure 7C and 7F respectively. In order to validate the model, we have compared our results with the gene interactions known in literature or extracted from the REGULON DB database [31].

The SOO method identified a number of gene connections that were known to play a role in acid adaptation. These included the interaction between two of the glutamate-dependent acid-stress response genes gadW and gadX [32]. However, in this model the directions of the gene interactions are mostly incorrect and not representative of the known *E. coli *acid response mechanisms. For example, the coding glutamate decarboxylase gene gadB is unlikely to be involved in the modulation of the two-component system PhoP/PhoQ.

On the contrary, the gene regulatory network derived from the application of the MOO procedure (Figure 7D) includes several gene interactions known to be important in acid adaptation.

A key interaction involved the two-component system PhoP/PhoQ [33]. This complex is a known upstream regulator of acid adaptation. The model we developed (Figure 7C) reflects the upstream regulatory role of this complex and correctly predicted its influence in the regulation of the acid resistance genes gadW and hdeA [34]. The network also shows the known negative interaction between gadX and gadW [32] and the inhibition of the crp gene by fis [35,36]. Another validated interaction found by the MOO procedure is represented by the link between the histone-like protein hns and cadA [37]. Our model shows that hns may activate the expression of cadA. The connection is consistent with the literature, however, in GNB7145K hns mutants Shi et al. [37] have shown that hns acts as a repressor.

Some of the interactions in the network represent potentially novel regulatory mechanisms in *E. coli *response to acid stress. An example is the hypothesis that phoP may be involved in the activation of narX, a nitrite/nitrate sensor protein. This is a gene that is part of a two-component system regulating a component of anaerobic metabolism, which is a function known to be regulated during acid response [38].

## Discussion

In this paper we presented the gene regulatory network inference method "Network Inference with Multi Objective Optimization" (NIMOO).

When tested on simulated and "real world" datasets, NIMOO performs well even if incomplete data are available. The main feature of this methodology is that it can be used to develop dynamical models of gene regulatory networks integrating multiple data sources and combining any existing network inference methodology to identifying the network topology.

Although other methods have the potential to include prior knowledge in the inference process the ability to plug-in different inference methods in the same modelling procedure is so far a unique feature of NIMOO. In this paper we tested this concept and demonstrated that the approach can be successful even if a relatively simple procedure is integrated in the ODE model parameter estimation. However, a more comprehensive testing may be required to explore the full potential of this approach, for example combining more advanced methods in the MOO optimization procedure.

In terms of data integration, we have mainly focused on gene KO experiments. However, some of the procedures we have tested (e.g. MOO-Tc and MOO-Sc) are directly applicable to other types of experimental data. For example, a compendium of environmental and growth factor-induced perturbations could be employed to develop an objective compatible with these procedures. Such objectives could be for example computed by using the information theoretical approach ARACNE [5].

Moreover, additional information, for instance the confidence level in transcription factor binding consensus sequences in a gene's promoter region could also be incorporated within the optimisation process. More generally, in the event that multiple objectives are used within a MOO procedure, each function's relative importance could be weighted by adjusting the optimization parameters, such as weights θ_k _(Equation 4). Additionally, any definite qualitative knowledge of the presence or absence of gene connections may be used as a constraint on the inferred gene-regulatory matrix (hard prior).

Because of the ability to integrate different methods the user can very easily customize NIMOO. In this respect, NIMOO is an integration framework rather than a specific method. Comparing its performance with existing methods is therefore not necessarily consequential. However, we have performed an initial assessment comparing some implementations of NIMOO to other methods. For example, all NIMOO procedures outperformed TSNI [10] in inferring undirected networks (Table S1 in Additional File 1) and the MOO-S_ens _and MOO-T_ens _performed better with both undirected and direct-signed networks (Table S1 in Additional File 1). Moreover, NIMOO performed in a comparable manner to the method developed by Yip et al. [15], which won the DREAM3 competition http://wiki.c2b2.columbia.edu/dream/index.php/ (Table S2 in Additional File 1).

So far the application of multi-objective optimization methods to inferring biological networks has been limited: For example, van Someren et al. [39] and Fomekong-Nanfack et al. [40] used MOO to incorporate multiple constraints arising from the requirement of stability and robustness of gene networks, and, Liu and Wang [41] have used MOO to infer biochemical networks by simultaneously minimizing for the concentration error and the slope error. However, in all these cases a single data set and a single reverse engineering criterion were used.

## Conclusions

The network-inference framework NIMOO is flexible and can be used in many different scenarios, even when available information is incomplete. The application of NIMOO to biological datasets representing two different "real world" scenarios produced very interesting results. The analysis of the experimental datasets illustrated that inclusion of additional objectives from the same dataset could significantly improve our ability to identify key regulators of relevant biological processes.

## Authors' contributions

RG implemented the method, performed the validation and contributed to the writing of manuscript. PA contributed with the processing and analysis of the experimental data. AS and SD performed the microarray experiments. AB and RB designed the tumor implantation experiments and AB performed the experiment. FF designed the approach and contributed to writing the paper. All authors read and approved the final manuscript.

## Supplementary Material

Additional File 1 **A method comparison study and additional tables and figures as detailed in the body of the paper**. Click here for file
