# Supplementary material for: A computational framework for gene regulatory network inference that combines multiple methods and datasets
Source: BMC Syst Biol. 2011 Apr 13;5:52. doi: 10.1186/1752-0509-5-52 (PMC3098160; doi:10.1186/1752-0509-5-52)
Supplement: Additional File 1 — A method comparison study and additional tables and figures as detailed in the body of the paper. [file 1752-0509-5-52-S1.PDF]

SUPPLEMENTARY MATERIAL

A Computational Framework for Gene Regulatory Network Inference that Combines Multiple Methods and Datasets

Rita Gupta[1], Anna Stincone[1], Philipp Antczak[1], Sarah Durant[2], Roy Bicknel[2], Andreas Bickfalvi[3] and Francesco Falciani[1]

[1] School of Biosciences, University of Birmingham, Edgbaston, Birmingham B15 2TT, UK. [2] Institute of Biomedical Research, Medical School, University of Birmingham, Edgbaston, Birmingham B15 2TT, UK. [3] INSERM E 0113, Molecular Angiogenesis Laboratory, Université de Bordeaux 1, 33405 Talence, France.

A comparison between NIMOO and TSNI

In order to compare the performance of the methodology we developed with some of the top performing methods available in the public domain we first set to benchmark NIMOO with "Time Series Network Inference" (TSNI), a modelling approach developed by Bansal et al. [1]. TSNI infers direct-signed gene networks from microarray time series data using an ODE model coupled with a dimensionality reduction technique to reduce the complexity of the problem.

In this paragraph we describe the results of applying TSNI to the GNW networks used to evaluate NIMOO and their comparison with the performance of the SOO, D-Sp, MOO-DSp, MOO-S_{ens} and MOO-T_{ens} procedures (**Table S1)**.

All procedures, including SOO outperform TSNI for undirected networks (**Table S1**). For direct-signed networks, the procedures that integrate gene KO experiments (MOO-S_{ens} and MOO-T_{ens}) outperform TSNI for both undirected and direct-signed networks (**Table S1**). However, TSNI outperforms SOO and MOO-DSp procedures for direct-signed networks. Interestingly, a relatively simple time delay correlation matrix (DSp) performs similarly to TSNI in predicting direct-signed networks.

Type	Size	SOO	DSp	MOO-DSp	MOO-S_{ens}	MOO-T_{ens}	TSNI
Undirected	20	0.68	0.77	0.79	0.77	0.70	0.51
Undirected	35	0.68	0.73	0.79	0.88	0.79	0.54
Undirected	50	0.65	0.68	0.76	0.85	0.79	0.51
Direct-signed	20	0.27	0.32	0.23	0.47	0.39	0.33
Direct-signed	35	0.27	0.30	0.22	0.69	0.57	0.31
Direct-signed	50	0.24	0.30	0.31	0.64	0.51	0.34

Table S1 Comparison between NIMOO and TSNI

The table shows the results of a comparison between several procedures implemented in NIMOO and TSINI. AUC values for the SOO, DSp, MOO-DSp, MOO-S_{ens} and MOO-T_{ens} procedures that are significantly ($p<0.05$) higher than AUC values obtained with TSNI are marked in red whereas AUC values that are significantly lower are marked in green. When there is no significant difference the AUC values are marked in black.

Comparison with the Yip et al. method

We have shown that overall NIMOO compare well or outperform TSNI in most cases, particularly when gene KO data is included in the analysis. Since TSNI does not use such information we reasoned it was important to compare NIMOO's performance with a method that has been specifically designed to integrate time course and gene inactivation data.

For this comparison we choose a method developed by Yip et al. [2]. Since this method won the DREAM3 competition we set to apply NIMOO to the time course and homozygous deletion data from the DREAM3 Challenge (website http://wiki.c2b2.columbia.edu/dream/index.php/), and, compare our results to those of Yip et al. [2].

The method by Yip et al. gives prime importance to the homozygous deletion data. We wondered whether our methodology would perform as well as that of Yip at al. under the same assumptions. Therefore, we make the same assumption that the homozygous deletion data is a good reflection of the gene network. This is achieved by adjusting the weights in our algorithm in favour of the steady-state matrix. **Table S2** shows that, under these conditions NIMOO accuracy is comparable with the AUC values reported by Yip et al. (**Table S2**). It is interesting to notice that the gene KO matrix computed with the ratio procedure described in this paper also achieve the same accuracy than NIMOO and the Yip et al method.

We have also applied SOO to the DREAM3 Challenge data (i.e. using time course data only). The AUC values for the networks ranged between 0.45 and 0.55. This figure is higher than those reported by Yip et al. when they apply their basic ODE method to the time course data (Yip et al. [2], Table 3 and Table 6).

Networks	E.Coli1	E.Coli2	Yeast1	Yeast2	Yeast3
**Network size: 10 genes**					
Yip et al.	0.93	0.91	0.95	0.75	0.75
GK	0.98	0.91	0.91	0.71	0.67
NI_MOO	0.96	0.89	0.91	0.74	0.71
**Network size: 50 genes**					
Yip et al.	0.93	0.92	0.92	0.79	0.81
GK	0.9	0.93	0.86	0.76	0.76
NI_MOO	0.92	0.93	0.86	0.75	0.75
**Network size: 100 genes**					
Yip et al.	0.95	0.96	0.92	0.86	0.78
GK	0.88	0.95	0.86	0.82	0.76
NIMOO	0.87	0.93	0.79	0.76	0.71

Table S2 Comparison of AUC values for the official direct-unsigned DREAM3 Challenge networks obtained by: 1) the method developed by Yip et al. [2], 2) the homozygous deletion matrix (GK), and 3) from NIMOO.
A Wilcoxon rank sum test comparing Yip et al's and NIMOO methods for the yeast networks gave a *p value* of 0.4, 0.3 and 0.2 for the 10-genes, 50-genes and 100 gene networks, respectively.

Additional Figures and Tables

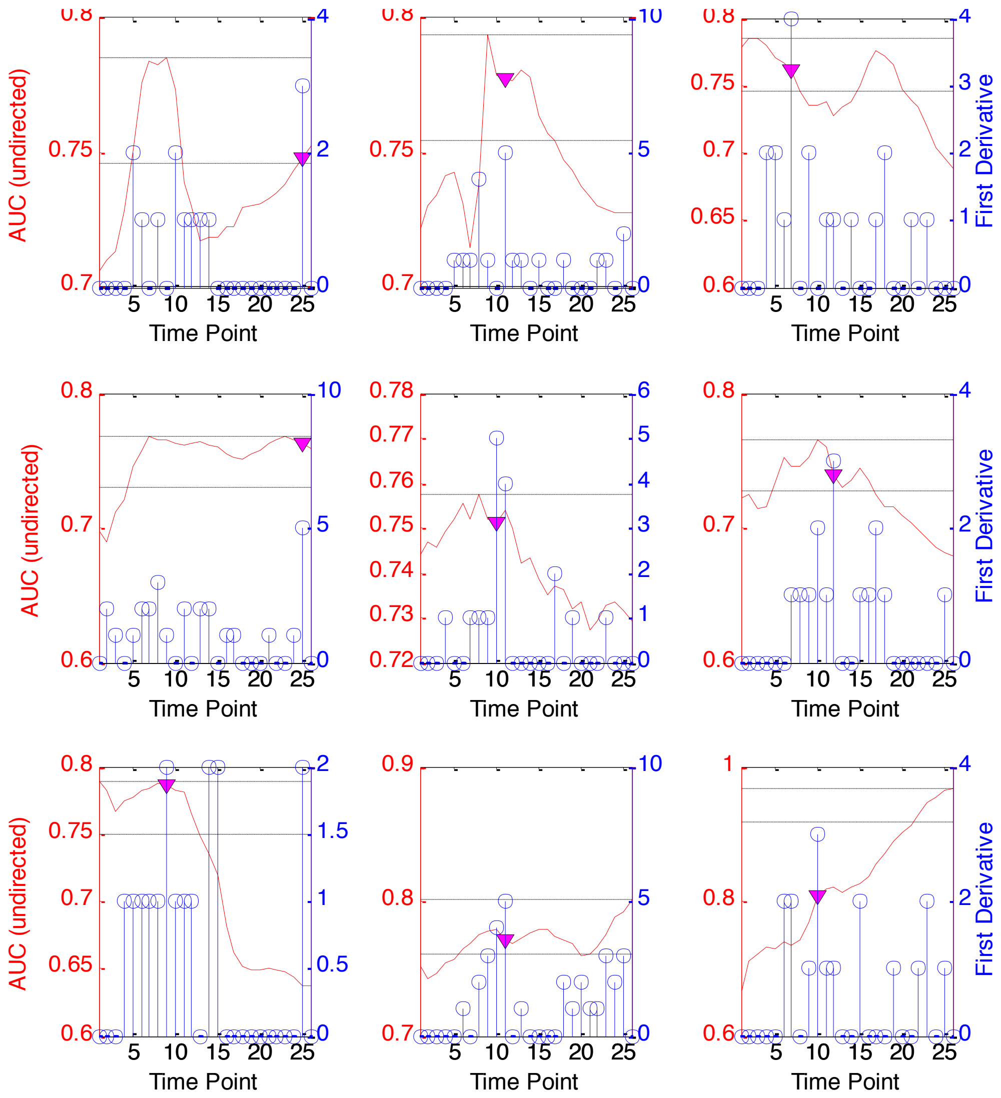

Figure S1 Influence of t_p on inference accuracy in MOO-Tr procedures.

The figure represent the The y-axis scale on the left side indicates the AUC values correspondent to inference of undirected networks. The y-axis on the right side of each plot (blue scale) represent the number of gene expression profiles with maximum first derivative at a given time point (x-axis). Dashed horizontal lines indicate 5% interval below the maximum AUC value, which is indicated by the filled inverted triangle

.

Gene symbol	Fold Change (log_2)	Adj p value	Gene symbol	Fold Change (log_2)	Adj p value
ANXA6	2.443422	0.004061	JAK2	2.836042	0.005718
BIRC3	3.863097	0.069638	LAMA4	11.37413	9.76E-07
BMP1	6.561977	0.000106	LUM	4.577634	0.002162
CASP3	1.577769	0.021921	MMP9	5.028760	0.006540
CASP7	3.279089	0.005008	MSN	2.133074	0.000423
CAV1	2.409116	0.001857	NFE2L2	1.647569	0.056845
CDC42	1.799494	0.017911	NFKBIA	4.529538	0.039824
CDGAP	2.373746	0.004358	PAK4	2.60589	0.008007
CLEC3B	6.074838	0.000706	PDGFRB	7.503855	0.000108
COL1A2	16.9809	1.35E-06	PLAT	3.479982	0.0004
COL3A1	42.37015	2.86E-07	PLAU	5.092899	0.00031
COL4A2	4.038535	2.84E-05	PSEN2	1.729793	0.007959
COL5A2	21.08291	3.73E-06	PTEN	1.747071	0.217235
COL5A3	28.88583	5.56E-07	PTPN13	2.650549	0.000252
CRK	1.566122	0.473731	RAC1	1.313549	0.58563
DLST	1.423828	0.392728	RALBP1	1.626825	0.034923
DOCK1	1.94526	0.000384	RCC2	2.966488	0.000721
DOK4	2.192999	0.016008	RHOC	2.004674	0.005796
ERBB”	1.589950	0.075840	ROCK1	2.105359	0.13288
F2R	3.696626	0.002685	SDC2	6.259481	9.58E-05
FAM46A	2.398403	0.035232	SERPINE2	4.62354	0.00852
GJB2	3.948322	0.019359	SPARC	7.090896	6.27E-05
GOLGA3	1.889651	0.026367	SYNJ2	1.884404	0.196865
HSPB1	2.746557	0.020032	TERF1	1.536759	0.485853
ITGA2	5.622236	0.000509	TIAM!	2.40215	0.001305
ITGA5	2.221099	0.018222	TGFBI	4.977775	0.000361
ITGAV	3.926613	0.000133	VCAM1	7.150623	0.0007
ITSN2	2.615156	0.032181	ZHX1	2.0251	0.00483

Table S3 List of genes selected for modeling in vivo tumor development

Gene symbols are indicated alongside the fold chance expressed as a logarithm of the ratio between maximum and minimum expression across the time course. Adjusted *p values* (Benjamini-Hochberg correction) are also reported.

References

1. Bansal, Gatta, Di Bernardo (2006) Inference of gene regulatory networks and compound mode of action from time course gene expression profiles. BioInformatics **22**, 815-822.
2. Yip KY, Alexander RP, Yan K-K, Gerstein M (2010) Improved Reconstruction of *In Silico* Gene Regulatory Networks by Integrating Knockout and Perturbation Data. PLoS ONE 5(1): e8121. doi:10.1371/journal.pone.0008121
